# ReactionT5: a pre-trained transformer model for accurate chemical reaction prediction with limited data

**DOI:** 10.1186/s13321-025-01075-4

**Published:** 2025-08-19

**Authors:** Tatsuya Sagawa, Ryosuke Kojima

**Affiliations:** 1 Graduate School of Pharmaceutical Sciences, Kyoto University, Kyoto, 606–8501 Japan; 2 Graduate School of Medicine, Kyoto University, Kyoto, 606–8501 Japan; 3 RIKEN BDR, Kobe, 650-0047 Japan

**Keywords:** Foundation model, Chemical reaction, Reaction space, Deep learning, Machine learning, Transformers, Organic chemistry

## Abstract

Accurate chemical reaction prediction is critical for reducing both cost and time in drug development. This study introduces ReactionT5, a transformer-based chemical reaction foundation model pre-trained on the Open Reaction Database—a large publicly available reaction dataset. In benchmarks for product prediction, retrosynthesis, and yield prediction, ReactionT5 outperformed existing models. Specifically, ReactionT5 achieved 97.5% accuracy in product prediction, 71.0% in retrosynthesis, and a coefficient of determination of 0.947 in yield prediction. Remarkably, ReactionT5, when fine-tuned with only a limited dataset of reactions, achieved performance on par with models fine-tuned on the complete dataset. Additionally, the visualization of ReactionT5 embeddings illustrates that the model successfully captures and represents the chemical reaction space, indicating effective learning of reaction properties.

**Graphical Abstract:**

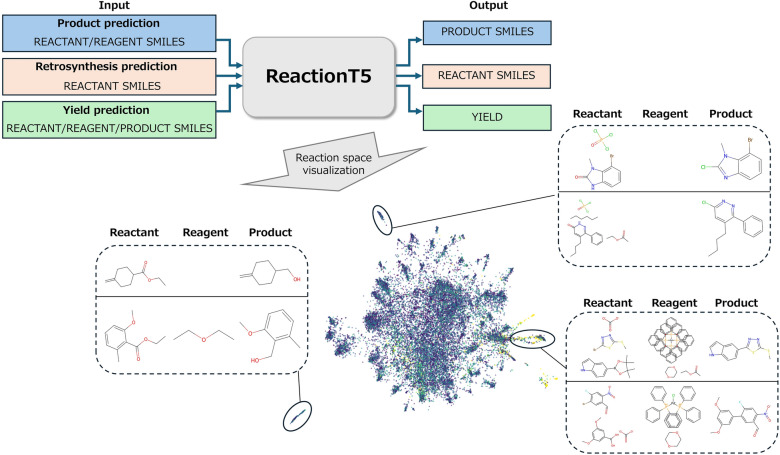

**Supplementary Information:**

The online version contains supplementary material available at 10.1186/s13321-025-01075-4.

## Scientific contribution

We propose ReactionT5, a foundation model for chemical reactions pre-trained on a large reaction dataset that outperforms existing models in product prediction, retrosynthesis, and yield prediction tasks, all while maintaining high performance even with limited training data. We also show that the learned embeddings effectively capture the chemical reaction space, and we make the training code and model weights publicly available. We believe that fine-tuning ReactionT5 with users’ in-house data will be beneficial for many chemical applications.

**Supplementary Information:**

The online version contains supplementary material available at 10.1186/s13321-025-01075-4.

## Introduction

Predicting chemical reactions is pivotal for progress in organic chemistry and drug discovery. Highly accurate predictive models can dramatically reduce the costs associated with exploratory experimentation by forecasting the outcomes of chemical experiments before they are conducted. Consequently, machine learning models that adeptly encapsulate the complexity of organic reactions [1] are anticipated to bolster the efforts of experimental chemists. Deep learning models have surfaced as viable substitutes in recent years, providing data-driven insights derived from extensive reaction datasets and surmounting numerous constraints of traditional methods [2].

In this context, large-scale pre-trained models utilizing compound libraries have attracted increasing interest in organic chemistry research [3]. These models often conceptualize molecules as symbolic sequences, similar to natural languages. For instance, SMILES-BERT [4] has demonstrated high efficacy in predicting molecular properties by leveraging unsupervised pre-training on molecular structures encoded in the Simplified Molecular-Input Line-Entry System (SMILES) format [5]. While substantial progress has been made with models focused on single molecules, for example, SMILES-BERT [4], ChemBERTa [6], MolE [7], MolCLR [8], and Molformer [9], research on models addressing multiple molecules, such as those used in chemical reactions, remains relatively scarce. A notable exception is T5Chem [10], which, although not explicitly pre-trained on reaction datasets, has shown potential as a reaction prediction model by pre-learning from single molecules and subsequently applying fine-tuning to address multiple tasks, including product, retrosynthesis, and yield prediction. Building upon these advances in molecular representation learning, recent studies have extended pre-training approaches to chemical reactions using reaction SMILES, which are chemical transformations represented in SMILES format. Pre-training on reaction data has been shown to enhance model performance on various downstream tasks, such as yield prediction [11] and experimental procedure prediction [12], highlighting the importance of capturing the intricate dependencies within chemical reactions.

Chemists anticipate that foundation models for chemical reactions will perform well on target tasks with minimal fine-tuning using a small set of reaction data, as accumulating a large volume of experimental data for training is challenging. This need is particularly pronounced when there is a domain shift between the datasets used for pre-training and those intended for the target tasks, highlighting a critical demand for developing models based on chemical reactions that can be efficiently and effectively fine-tuned with limited data. To our knowledge, there are currently no pre-trained models specifically designed to be fine-tuned on small data sets that have been pre-trained using a large-scale reaction database.

This study introduces ReactionT5, a chemical reaction foundation model that utilizes the text-to-text transfer transformer (T5) [13] architecture. ReactionT5 is distinct in that it incorporates pre-training on a vast reaction database in addition to the conventional pre-training on a molecular library. By leveraging the Open Reaction Database (ORD) [14], an expansive, open-access dataset covering a broad reaction spectrum, as our pre-training database, we aim to broaden the reaction space the model can capture. Our goal is to provide a solution that remains effective for downstream applications, even with minimal data for fine-tuning.

In this study, we evaluate ReactionT5 on downstream tasks such as product prediction, retrosynthesis, and yield prediction, while also using reaction space visualization to better understand the model’s behavior in their tasks. Visualization of chemical reaction space has recently gained attention as a powerful tool for capturing similarities and diversity among reactions. Previous studies have attempted to visualize reaction embeddings obtained from trained models to explore the chemical knowledge learned by the models [15, 16]. Following these ideas, we also visualized the embedding of ReactionT5 to confirm the chemical reaction space that this model captures. Such visualizations are expected to assist chemists in identifying clusters of similar reactions, discovering outliers, and ultimately discovering novel reactions.

The contributions of this paper are outlined as follows:

- 1. We evaluated the effectiveness of utilizing an extensive reaction database like the ORD for model pre-training.
- 2. We showed that our chemical reaction foundation model achieves high performance, even with limited fine-tuning data.
- 3. We generated reaction embeddings from the model and visualized the reaction space to illustrate its understanding.

These contributions demonstrate that ReactionT5 exhibits strong generalizability and excels in real-world scenarios with limited fine-tuning data. This establishes ReactionT5 as a promising and practical tool for a wide range of applications in the field.

## Methods

**Fig. 1:**
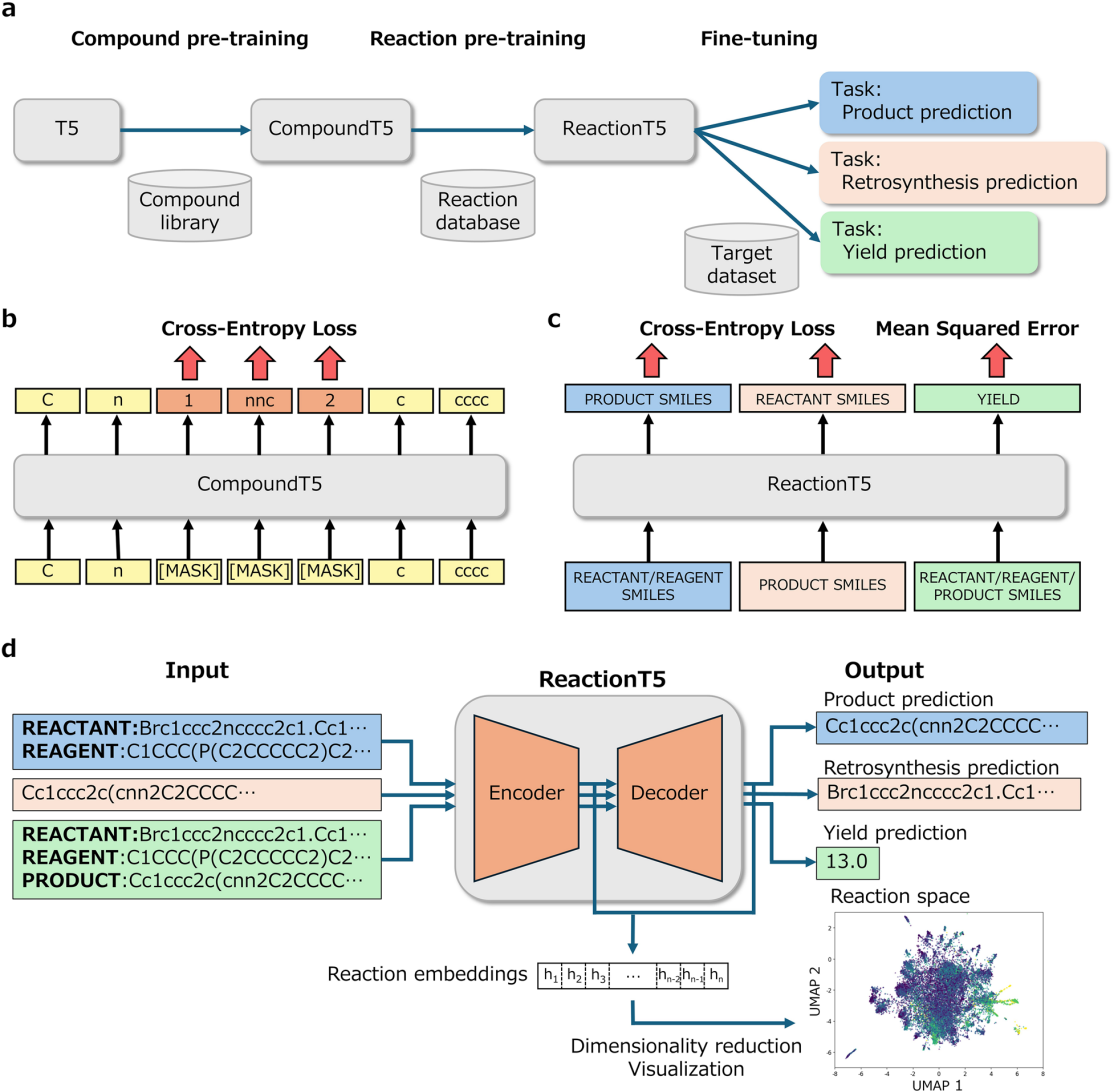
**a** Workflow of our method: the process begins with the base T5 model, which is pre-trained on a compound library to create CompoundT5. Next, ReactionT5 is developed by further pre-training CompoundT5 on a reaction database. Finally, ReactionT5 is fine-tuned on relatively small datasets, such as in-house data. **b** Illustration of compound pre-training: CompoundT5 is trained using a masked language modeling objective, where the task is to predict masked tokens in SMILES strings representing individual compounds. **c** Illustration of reaction pre-training: ReactionT5 is trained to perform three types of tasks: predicting product’s SMILES from reactants and reagents, predicting reactant’s SMILES from products, and predicting reaction yield from reactant, reagent, and product. **d** Diagram of ReactionT5 usage: ReactionT5 processes input sequences in text format, incorporating special tokens within the SMILES strings to denote the roles of reactants, reagents, and products

This study utilized the T5 model as the neural network architecture and implemented a two-stage pre-training followed by task-specific fine-tuning. The initial framework and methodology are depicted in Fig. 1a, which illustrates our two-stage pre-training process: compound pre-training and reaction pre-training. Initially, in the compound pre-training stage, the T5 model was trained on a library of single-molecule structures, resulting in a specialized model called CompoundT5. Subsequently, in the reaction pre-training stage, we expanded CompoundT5’s capabilities by training it on a reaction database encompassing chemical reaction data involving multiple compounds, such as reactants, reagents, catalysts, solvents, and products. This training phase enabled the model to capture the intricate relationships between the compounds within each reaction, culminating in the creation of ReactionT5. Upon completing these pre-training stages, ReactionT5 was then ready to be fine-tuned on smaller, task-specific target datasets.

Figure 1d illustrates the operational flow from input to output for ReactionT5. In this setup, chemical reactions are encoded as textual sequences in the SMILES format, positioning ReactionT5 within a text-to-text framework. This structure harnesses the capabilities of the T5 architecture, which is explicitly designed for text-to-text conversions, making it ideally suited for handling reaction-related tasks as text-based challenges. ReactionT5 consists of two main components: an encoder that transforms the input text into hidden representations and a decoder that generates the output text based on these representations. This methodology was applied to three critical tasks: product prediction, retrosynthesis, and yield prediction, showing the model’s versatility and effectiveness in handling complex chemical data.

### Compound pre-training

In the first pre-training stage, we developed CompoundT5 by pre-training an initialized T5 model on a dataset of compounds encoded in the SMILES format. The preparation for training involved tokenization, a process where input text is segmented into smaller tokens corresponding to subwords in the SMILES text. Following tokenization, we implemented span-masked language modeling (span-MLM) [13], a self-supervised learning technique wherein the T5 model is trained using only input texts. In span-MLM, contiguous sets of tokens (spans) within the input SMILES text are masked, and the model is tasked with predicting these masked spans. This differs from the traditional masked language modeling (MLM), which involves masking and predicting individual tokens. Span-MLM presents a more challenging scenario requiring the model to interpret broader contextual information [17], thereby enhancing its ability to understand molecular structures from SMILES representations.

For training CompoundT5, we adhered closely to the procedures outlined in the original T5 paper [13]. We employed span-MLM by randomly masking 15% of the tokens in the input text, with the average length of masked sequences being three tokens. We utilized a SentencePiece unigram tokenizer [18], specifically trained on our compound library. Compared to character-level or atomic-level tokenizers, this tokenizer efficiently represents compounds using fewer tokens, accelerating training and inference processes and allowing for the inclusion of larger molecular structures [19]. We trained Google's T5 Version 1.1 base model [13] for 30 epochs using a learning rate of 0.005, a weight decay of 0.001, and a batch size of 5, with the Adafactor optimizer [20]. All training was conducted on a single NVIDIA RTX A6000 GPU.

### Reaction pre-training

In the subsequent pre-training stage, known as reaction pre-training, we transition from a compound-level representation to representing all chemical reactions as text. This stage differs from the compound pre-training, where each compound was independently transformed into a textual format. In reaction pre-training, the entire reaction, as extracted from the reaction database, is converted into a single textual input (as depicted in Fig. 1d). This approach allowed the CompoundT5 model to be further trained to grasp the contextual intricacies of each reaction extending beyond the individual compound components. This comprehensive pre-training aimed to prepare the model for three critical tasks: product prediction, retrosynthesis prediction, and yield prediction (Fig. 1c).

The reaction records from the reaction database used in our study include information corresponding to six roles: reactant, reagent, solvent, catalyst, product, and yield. Compounds associated with the first five roles are encoded in the SMILES format, whereas yield is represented as a numerical value. In scenarios where multiple compounds share the same role, their SMILES strings are concatenated using a “.” token to denote separation. Special role tokens are incorporated into the input sequence to enhance clarity in the roles of each compound within the reaction. For example, accurately distinguishing among catalysts, reagents, and solvents can be challenging in real-world datasets; hence, these three roles are collectively labeled under the “reagent” category. Special tokens such as “REACTANT:” and “REAGENT:” are prepended to their respective SMILES sequences to further differentiate reactants from reagents. Finally, the sequences are concatenated into a single string with labeled roles, forming the complete input text to train the model.

The reaction pre-training uses the same objective functions as those used in downstream tasks such as product, retrosynthesis, and yield prediction. The difference lies in the use of a large-scale reaction database prior to task-specific fine-tuning. This pre-training allows the model to learn broad reaction space representation in advance, thereby enabling improved performance even with limited fine-tuning data. The specific configurations for each task are described in Sect. 2.3 to 2.5.

### Product prediction

The product prediction task, also known as forward prediction, entails predicting the products of a reaction from given compounds, specifically from the roles of reactants and reagents as encoded in SMILES text. In this task, ReactionT5 aims to forecast the reaction products based on the input SMILES text. The decoder of the T5 model is pivotal in ensuring the accuracy of the generated text representing products. It computes a probability distribution over the vocabulary of tokens, iteratively predicting the subsequent tokens based on the encoder's outputs and previously generated tokens. During each decoding step, the decoder selects tokens to maximize the probability of the entire sequence, concluding when an end-of-sequence token is generated. The probability of the complete sequence is calculated as the product of the probabilities of the selected tokens.

Given that exhaustively evaluating all possible token combinations to identify the sequence with the highest probability is computationally prohibitive, a beam search strategy is employed to streamline the search process. The model efficiently retrieves the Top-5 most probable product predictions by setting the beam width to five. For optimization, our experiments use the average token-level cross-entropy as the loss function.

### Retrosynthesis prediction

Retrosynthesis prediction involves a text-to-text conversion task, operating in the reverse direction of the product prediction task. This task entails predicting the reactants from given products, aligning with the methodologies of prior research. Unlike product prediction, this task does not require the prepending of special tokens to the products in the input. This is because the input solely consists of product compounds, and the model does not need to explicitly differentiate these product compounds from other types of compounds.

### Yield prediction

The yield prediction task involves forecasting the numerical yield value based on the molecules of reactants, reagents, and products. For this task, special tokens “REACTANT:”, “REAGENT:”, and “PRODUCT:” are prepended to the respective SMILES sequences, which are then concatenated to create a unified input sequence. During pre-processing, any yield values exceeding 100% are clipped to 100%, and all values are rescaled to a 0–1 range. This clipping is based on the observation that yields exceeding 100% often result from experimental noise, such as solvent inclusion, byproduct incorporation, or measurement error, rather than reflecting true chemical outcomes [21]. Although such values can occur in practical settings, their frequency in the dataset is very low (0.271%). To prevent extreme values from exerting an undue influence on model training, we applied clipping to yields above 100%, rather than discarding these samples. To predict the yield, embeddings from both the encoder and decoder are fed through four additional fully connected layers, which output the numerical yield predictions. Mean squared error is used as the loss function to minimize the differences between predicted and actual yields.

### Evaluation

We assessed the performance of ReactionT5 across the different tasks using benchmark datasets and compared the results with those of conventional methods.

We report Top-k accuracies (k = 1,2,3,5) and the Top-5 SMILES invalidity rate for product and retrosynthesis prediction. A prediction is considered accurate if its canonicalized SMILES string exactly matches the reference label, and Top-k accuracy is defined as the proportion of reactions where the correct answer is among the Top-k predictions. Additionally, we calculated the SMILES invalidity rate, which measures the fraction of invalid SMILES strings within the Top-5 predictions, using RDKit to verify the validity of the SMILES strings. The model outputs the five most probable compounds, which are then canonicalized using RDKit.

In yield prediction, the performance is measured using the coefficient of determination (R^2^), which assesses the correlation between the predicted and actual yields. Values closer to 1 indicate higher predictive accuracy.

We also addressed potential data leakage by identifying and removing any duplicate reactions in both the training and evaluation datasets to ensure a fair and unbiased evaluation. This step was taken to prevent the model from simply memorizing specific reactions, thereby ensuring that the evaluation genuinely reflects the predictive capabilities of the model rather than its capacity to recall seen data.

## Datasets

### Compound library

For the compound pre-training stage, we utilized a dataset of 24 million compounds from the ZINC20 database [22], with each molecule represented in SMILES format. All SMILES representations were canonicalized during pre-processing using RDKit (version 2023.9.6) to ensure consistency and standardization across the dataset. The character distribution within this dataset is depicted in Fig. S1a. The dataset was randomly split into two parts: 90% for training and 10% for validation.

### Reaction database: ORD

We employed a dataset of 1.5 million records from the ORD for reaction pre-training, accessed on June 5, 2024. This dataset features various reaction conditions, encompassing benchtop experiments, automated high-throughput setups, and flow chemistry techniques. To develop a model that operates independently of atom mapping, we pre-processed reactions containing atom-mapping information by removing these mappings. Moreover, we ensured the consistency of all SMILES strings across datasets by canonicalizing them.

To address biases related to the order of molecular presentation in ORD — specifically, the issue that leading reactants in the database are not always presented first, depending on the original data providers — we applied a canonicalization to the compounds. This introduces a degree of randomness, mitigating position-based biases that could impede model fine-tuning and broader usability. While the ORD contains various reaction-related details, including information about measuring devices or reaction conditions, we focused on extracting specific roles such as reactants, reagents, solvents, catalysts, products, and yield. The ORD aggregates publicly available reaction data, including commonly used benchmark datasets such as USPTO [23] and high-throughput experimentation (HTE) data. To avoid data leakage during evaluation, we carefully removed any reactions from the ORD that overlap with the test sets used in downstream tasks. Following pre-processing, we divided the dataset into three parts: 80% for training, 10% for validation, and 10% for testing.

A comparison between the reaction database and the compound pre-training dataset indicated a higher diversity of characters in the reaction database (as shown in Fig. S1). This diversity sometimes led to challenges, particularly with the tokenizer trained on the compound dataset, which occasionally failed to recognize certain atoms from the reaction database. This issue was notably significant with metal atoms such as “Pd” and “Fe”, which are commonly used as catalysts in chemical reactions but were not included in the compound dataset. As a result, model performance was adversely affected, as shown in Table S3 and Table S4. To address this problem, we extended the tokenizer vocabulary by incorporating additional tokens that appear frequently in reaction SMILES but were absent in the compound dataset. This extension allowed the model to correctly process important chemical symbols, including various metal atoms and reaction-specific characters, and thereby improved its handling of reaction data. A complete list of the added tokens is provided in Table S5.

### Target dataset

This section outlines the three target datasets corresponding to each task—product prediction, retrosynthesis prediction, and yield prediction—for fine-tuning and evaluation. These datasets are benchmarks for comparing ReactionT5 with other methods and assessing the model’s effectiveness in scenarios where only limited data are available for each task.

For the product prediction task, we employed the USPTO_MIT dataset [24], derived from Lowe’s patent data [23]. This dataset comprises 479,035 reaction records. In alignment with previous study [10], we divided the dataset into training (409,035 reactions), validation (30,000 reactions), and testing (40,000 reactions) datasets to ensure a comprehensive evaluation across different stages of model performance.

For retrosynthesis prediction, we used the USPTO_50k dataset [25], also based on Lowe’s patent data [23]. This dataset contains 50,000 reaction records, each featuring a single product. Consistent with established practices in prior research [10], the dataset was segmented into training (40,000 reactions), validation (5,000 reactions), and testing (5000 reactions) sets.

For yield prediction, the selected dataset was derived from the high-throughput experiments conducted by Ahneman et al. [26], focusing on palladium-catalyzed Buchwald-Hartwig C-N cross-coupling reactions. It includes 3955 reactions involving heteroaryl halides, 4-methylaniline, palladium catalysts, and various inhibitors. Following prior research [10], we approached the evaluation by randomly splitting the dataset into training and testing sets in a 7:3 ratio. This random division was repeated ten times to generate reliable statistics on the average performance and standard deviation. Additionally, four out-of-sample test sets (Test 1–4) were incorporated to assess the model's robustness and performance under conditions involving domain shifts.

## Results and discussion

In this section, we evaluate the performance of ReactionT5, which was pre-trained on ZINC20 and ORD datasets, across product prediction, retrosynthesis, and yield prediction tasks. We conducted a typical benchmark analysis, wherein the entire benchmark dataset was divided into training and evaluation segments for each prediction task. Additionally, a scenario involving limited data was employed to verify the impact of fine-tuning by utilizing only a small subset of the training data. We also visualized the reaction space represented by ReactionT5 to investigate how well the model captures the reaction space.

### Product prediction

For product prediction, the USPTO_MIT dataset served as the evaluation benchmark. In this analysis, we trained and evaluated ReactionT5 under conditions identical to those described in a prior study to determine its baseline performance (refer to Table 1).

**Table 1:** Top-k accuracy and Top-5 invalidity (%) of CompoundT5, ReactionT5, and conventional models in product prediction

	Top-1	Top-2	Top-3	Top-5	Invalidity
Seq-to-seq [27]	80.3	84.7	86.2	87.5	–
WLDN [28]	85.6	90.5	92.8	93.4	–
Molecular Transformer [30]	88.8	92.6	–	94.4	–
T5Chem [10]	90.4	94.2	–	96.4	-
CompoundT5	86.6	89.5	90.4	91.2	19.2
ReactionT5	**97.5**	**98.6**	**98.8**	**99.0**	**7.1**
ReactionT5 (zero-shot)	92.8	95.6	96.4	97.1	12.5

Table 1 provides a comparative analysis of CompoundT5 and ReactionT5 alongside several conventional methods: a sequence-to-sequence model (Seq-to-seq) [27], Weisfeiler-Lehman Difference Network (WLDN) [28] based on the Weisfeiler-Lehman Network [29], a transformer-based reaction prediction model (Molecular Transformer) [30], and a T5-based model (T5Chem) [10]. CompoundT5 refers to a model pre-trained solely on compound data (Fig. 1b), while ReactionT5 is initialized with the weights of CompoundT5 and further pre-trained on reaction data (Fig. 1c) as described in Sect. 2.3. Both models are subsequently fine-tuned on the training set from USPTO_MIT to evaluate downstream performance. The table also shows the performance of ReactionT5 in a zero-shot scenario, which is the original model without fine-tuning on USPTO_MIT, emphasizing its initial performance capabilities.

Moreover, to assess the effectiveness of ReactionT5 with limited fine-tuning data, we trained ReactionT5 and other models using small subsets of the dataset and evaluated their performance. ReactionT5 achieved a Top-1 accuracy of 97.5% in these experiments, surpassing T5Chem by 7.1 percentage points. Additionally, ReactionT5 demonstrated a significantly lower rate of invalid predictions compared to CompoundT5. The notably high zero-shot accuracy of ReactionT5, attributed to its pre-training on the ORD dataset, indicates that its predictive capabilities may match or surpass those of traditional methods, even without further fine-tuning on downstream datasets.

**Fig. 2:**
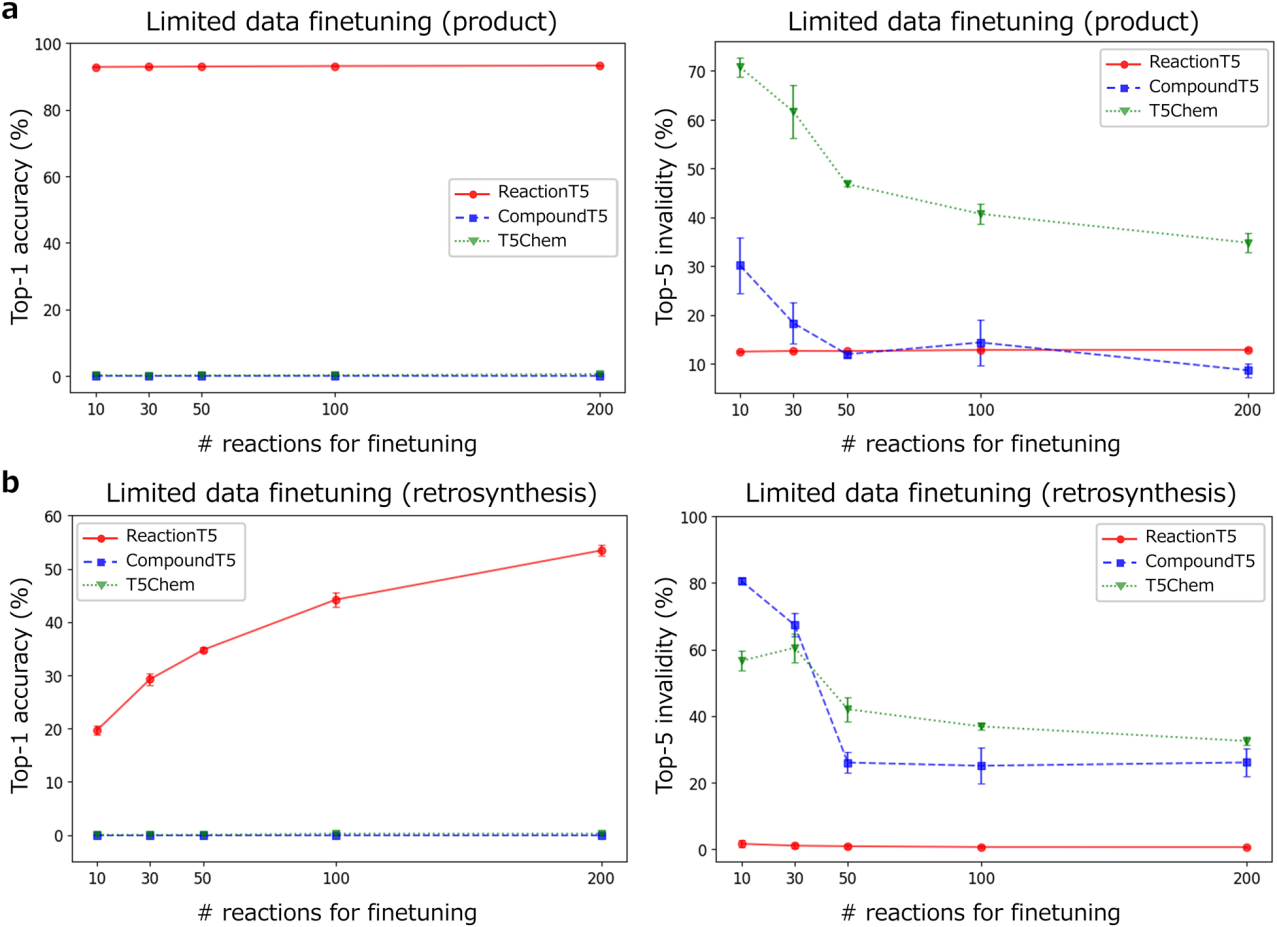
Model performance when fine-tuned with limited reaction data for product prediction (**a**) and retrosynthesis prediction (**b**) tasks. The left panels show changes in Top-1 accuracy, whereas the right panels depict variations in Top-5 SMILES invalidity rates as models are fine-tuned using subsets of 10, 30, 50, 100, or 200 reactions from the USPTO_MIT training set and evaluated on the testing set. The figures display the average Top-1 accuracy or Top-5 SMILES invalidity rate along with the standard deviation across the three experiments

To assess ReactionT5’s performance with minimal fine-tuning data, we fine-tuned ReactionT5, CompoundT5, and T5Chem using subsets of 10, 30, 50, 100, and 200 reactions from the USPTO_MIT training set, creating scenarios representative of scarce training data (Fig. 2a). For each subset size, reactions were randomly sampled three times, and the models underwent training three times. The average Top-1 accuracy and Top-5 SMILES invalidity rates, along with their standard deviations, were calculated based on these experiments. In line with its zero-shot prediction results, ReactionT5 exhibited high prediction accuracy and low invalidity rates, even with limited fine-tuning. Conversely, the other models faced challenges in generating reliable predictions, underlining ReactionT5’s robustness in data-constrained environments.

### Retrosynthesis prediction

For retrosynthesis prediction, we utilized the USPTO_50k dataset as the evaluation benchmark. Initially, we evaluated ReactionT5’s baseline performance (Table 2) and subsequently analyzed its adaptability in data-scarce conditions. This was done to understand how the model responds to limited fine-tuning data (Fig. 2b).

**Table 2:** Comparison of Top-k accuracy and Top-5 invalidity rates (%) for CompoundT5, ReactionT5, and conventional models in retrosynthesis prediction

	Top-1	Top-2	Top-3	Top-5	Invalidity
Seq-to-seq [27]	37.4	–	52.4	57.0	–
Molecular Transformer [30]	43.5	–	60.5	–	–
SCROP [31]	43.7	–	60.0	65.2	–
T5Chem [10]	46.5	–	64.4	70.5	–
CompoundT5	44.2	55.2	61.4	67.3	4.75
ReactionT5	**71.0**	**81.5**	**85.2**	**86.9**	**0.45**
ReactionT5 (zero-shot)	13.8	18.6	21.4	26.2	3.08

This table highlights the performance difference across models, showing the advantages of ReactionT5 in terms of both accuracy and reduced invalidity

Table 2 compares the benchmark performance of CompoundT5 and ReactionT5 with conventional methods, including Seq-to-seq [27], Molecular Transformer [30], T5Chem [10], and SCROP—a template-free self-corrected retrosynthesis predictor based on a transformer architecture with a syntax correction network [31].

ReactionT5 significantly outperformed other models, achieving a Top-1 accuracy of 71.0%, which is 24.5 percentage points higher than that of T5Chem. Additionally, ReactionT5 achieved a Top-5 accuracy of 87.9% and maintained a low Top-5 invalidity rate of 0.45%. These notable improvements are primarily attributable to the model’s extensive pre-training on the ORD dataset, which compensates for the limited size of the USPTO_50k training set, enabling the model to learn more effective retrosynthetic patterns. Although ReactionT5 demonstrated superior performance after fine-tuning, its zero-shot retrosynthesis accuracy was lower than that of zero-shot product prediction, likely due to dataset biases, such as the ORD reactions often involving multiple products, whereas the USPTO_50k dataset typically features single-product reactions.

Further evaluation was conducted to determine the minimal amount of data required for the model to adapt to dataset biases and to become applicable for downstream tasks. When ReactionT5 and other models were fine-tuned with limited reactions (Fig. 2b), the other models displayed no learning under these conditions. In contrast, ReactionT5 achieved a prediction accuracy of 46.1% when fine-tuned with just 100 reactions, nearly matching the accuracy of T5Chem trained on the full dataset.

### Yield prediction

**Fig. 3:**
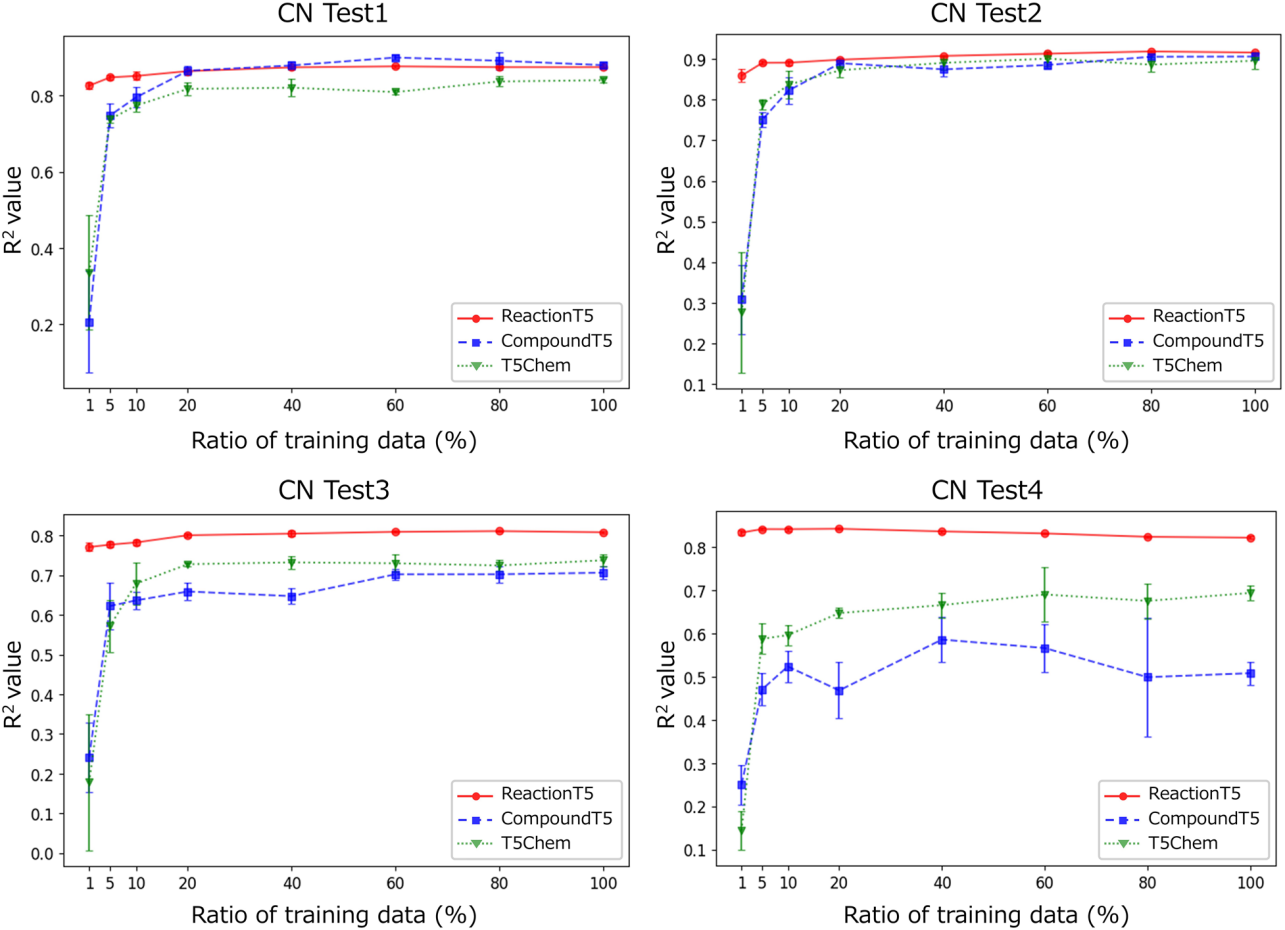
Model performance in yield prediction when fine-tuned with limited reaction data. The R^2^ values are shown for models fine-tuned with various fractions of training sets of each dataset. Training data was randomly sampled three times, and models were trained three times. The panels display the average R^2^ values along with the standard deviations across the three experiments

For yield prediction, we assessed ReactionT5 using a dataset of palladium-catalyzed Buchwald-Hartwig C-N cross-coupling reactions. This evaluation included both a benchmarking assessment (Table 3) and a small-data experiment (Fig. 3).

**Table 3:** Comparison of R^2^ for CompoundT5, ReactionT5, and conventional models in yield prediction

R^2^	Random 7:3	Test 1	Test 2	Test 3	Test 4	Avg. Test 1–4
DFT [26]	0.92	0.80	0.77	0.64	0.54	0.69 ± 0.10
MFF [32]	0.927 ± 0.007	0.851	0.713	0.635	0.184	0.596 ± 0.251
Yield-BERT [33]	0.951 ± 0.005	0.838	0.836	0.738	0.538	0.738 ± 0.122
T5Chem [10]	0.970 ± 0.003	0.811	0.907	0.789	0.627	0.785 ± 0.094
CompoundT5	**0.971 ± 0.002**	**0.889**	0.901	0.685	0.538	0.753 ± 0.151
ReactionT5	0.947 ± 0.003	0.875	**0.916**	**0.812**	0.819	**0.855 ± 0.043**
ReactionT5 (zero-shot)	0.831 ± 0.012	0.846	0.869	0.779	**0.843**	0.834 ± 0.034

CompoundT5 and ReactionT5 generally outperformed conventional models, with their performance varying based on the dataset type, highlighting their respective strengths

Table 3 compares the R^2^ values achieved by CompoundT5 and ReactionT5 with those of previously proposed models, including a random forest model with reaction descriptors for yield prediction (DFT) [26], a random forest model using multiple fingerprint features (MFF) [32], a BERT-based model with a transformer encoder and regression layer (Yield-BERT) [33], and T5Chem.

This outcome reveals that ReactionT5 retains robust predictive capabilities in yield prediction tasks. Specifically, while CompoundT5, fine-tuned on the C-N cross-coupling reactions within the training set, yielded the best performance on the randomly split dataset and Test 1, it underperformed in Tests 2–4. These tests presented more challenging domain-shift scenarios, a common struggle among conventional models. In contrast, ReactionT5 excelled under these difficult conditions, with its most significant improvements observed in Test 4, which previous studies have identified as the most challenging benchmark.

Furthermore, ReactionT5 demonstrated promising zero-shot performance, achieving superior results in Test 4 without additional fine-tuning.

We assessed ReactionT5’s performance in yield prediction under conditions of limited fine-tuning data by sampling fractions of the training sets from Tests 1–4 and training ReactionT5 alongside other models (Fig. 3). The training sets were randomly sampled, and the experiment was conducted three times. These results indicate that ReactionT5 consistently achieved high R^2^ scores across all scenarios, irrespective of the training data volume utilized. Specifically, ReactionT5 outperformed other methods, primarily when the data availability was restricted. While the other models required at least 20% of the training data (approximately 600 reactions) to reach their performance plateau, ReactionT5 demonstrated superior results, even excelling in zero-shot prediction scenarios.

### Visualization

**Fig. 4:**
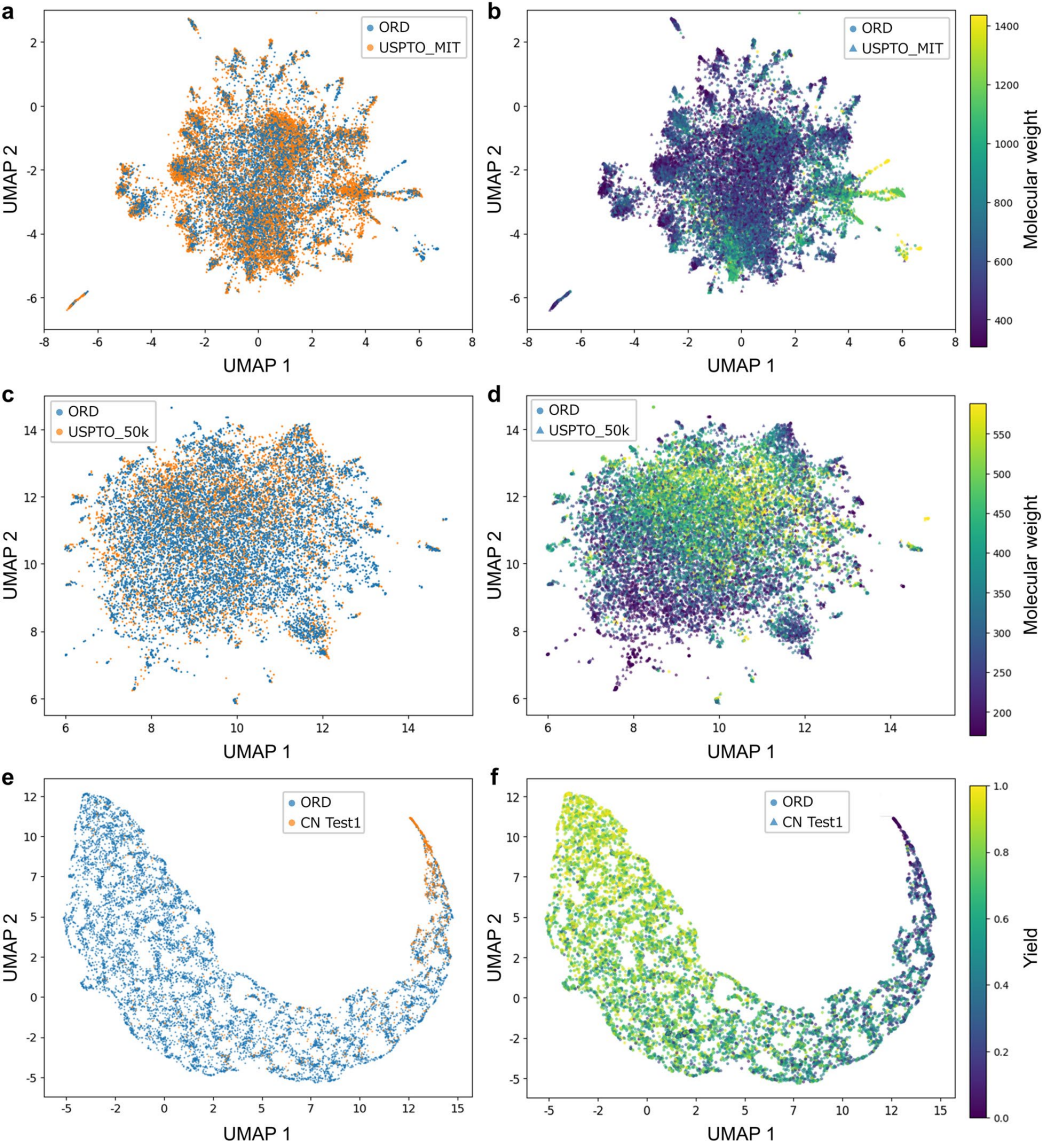
Visualization of the reaction space captured by ReactionT5 for product prediction (**a** and **b**), retrosynthesis prediction (**c** and **d**), and yield prediction (**e** and **f**). Input sequences for each reaction were encoded into hidden vectors and dimensionally reduced using UMAP. The left panels (**a**, **c**, and **e**) display the reaction space colored by dataset type, whereas the right panels (**b**, **d**, and **f**) depict variations based on either the molecular weight of input sequences or the reaction yield

Pre-training on an extensive and diverse reaction database likely enabled ReactionT5 to capture a broad reaction space. To verify this, we visualized the reaction space encoded by the model (refer to Fig. 1 for an overview of the process flow). Specifically, for product and retrosynthesis predictions, we extracted embeddings from the output of the encoder. We concatenated the embeddings from both the encoder and decoder for yield prediction to create a comprehensive reaction embedding. We then applied Uniform Manifold Approximation and Projection (UMAP) to reduce the dimensionality of these high-dimensional embeddings to two dimensions, facilitating the visualization of the reaction distributions in a two-dimensional space (Fig. 4).

Figure 4 displays the visualizations of the embedding space for each prediction task, with colors representing various chemical properties. Distinct clusters were observed within this figure, each correlating with trends in molecular weight and reaction yield. These patterns indicate that ReactionT5 effectively captures meaningful and relevant patterns within the reaction space, demonstrating the model’s sophisticated ability to represent and analyze complex chemical phenomena.

**Fig. 5:**
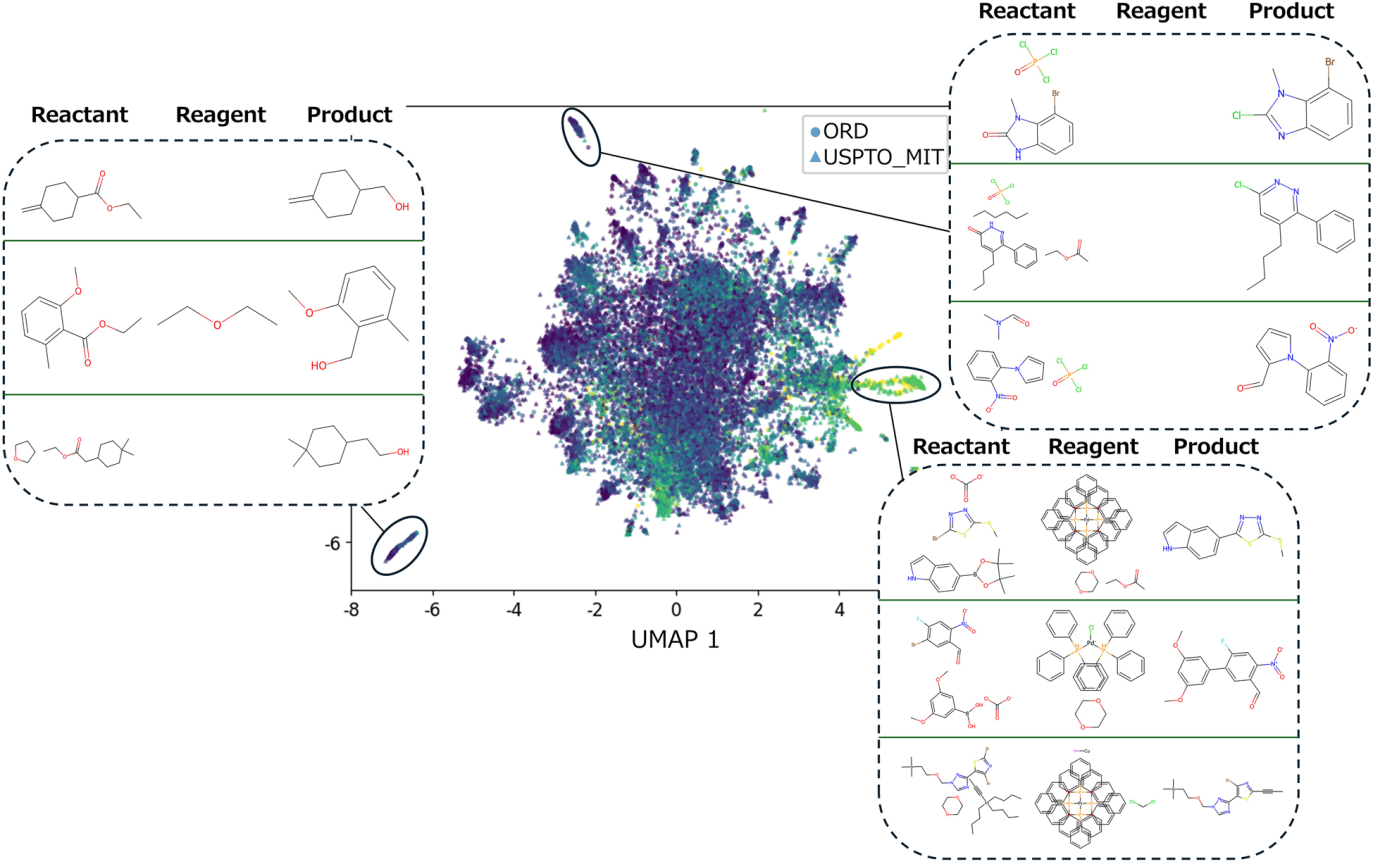
Qualitative analysis of ReactionT5’s understanding of the reaction space. Distinct regions in the reaction space revealed varying characteristics based on reaction types, compound architectures, and reaction conditions, highlighting the ability of the model to capture meaningful patterns and relationships within the reaction data

To qualitatively assess ReactionT5’s comprehension of the reaction space, we examined reactions situated in distinct areas of the 2D visualization and analyzed the chemical characteristics associated with these regions (Fig. 5). From Fig. 5, in the context of product prediction, reactions located in the lower-left region are predominantly hydrolysis of esters and reduction of carboxylic acids to alcohols. Conversely, the upper region is characterized by reactions involving nitrogen heterocyclic compounds, and the right region predominantly features reactions catalyzed by metal-phosphine complexes.

In retrosynthesis prediction (Fig. S3a), the reactions in the left region illustrate nucleophilic substitution reactions between carbon halides and nitrogen, the upper region is distinguished by reactions involving steroid ring-containing compounds, and the right region highlights reactions of compounds that contain silicon.

For yield prediction (Fig. S3b), the left region is defined by reactions involving compounds with 1,4-Dihydro-4-oxoquinoline-3-carboxylic acid as the parent structure, the middle region depicts nucleophilic substitution reactions between carbon halides and nitrogen, and the right region is characterized by reactions where a phosphorus-containing compound is used as a reagent.

These findings reveal that ReactionT5 has successfully captured and delineated the reaction space by identifying meaningful patterns and relationships within the reaction data, demonstrating its robust capability in mapping and interpreting complex chemical interactions.

We conducted a quantitative analysis to evaluate the quality of reaction embeddings generated by ReactionT5. In this experiment, we encoded all reactions from the ORD and target datasets using ReactionT5 into the designated reaction space. For each reaction in the target dataset, we employed the nearest neighbor method based on the embedding vector to compare the chemical reaction space, selecting the most similar reaction for prediction. The target datasets for products (product prediction) and reactants (retrosynthesis prediction) were identical to those used in the previous experiment. For yield prediction, we conducted evaluations on the Test 1–4 datasets.

For comparison, embeddings were generated using Morgan fingerprints (radius = 2 and embedding size = 1024) and T5Chem. For T5Chem, we used the model weights fine-tuned on Test 1–4 of the C-N cross-coupling reactions dataset, and their embeddings were generated by concatenating the embeddings from both the encoder and decoder. We assessed the prediction error by calculating the average Levenshtein distance between predictions and targets for product and retrosynthesis predictions. We then measured R^2^ for yield prediction.

Table 4 indicates that ReactionT5 achieved the lowest average Levenshtein distance for product and retrosynthesis predictions, as well as demonstrating a competitive R^2^ for yield prediction. These results demonstrate that ReactionT5 produces high-quality reaction embeddings and is an effective tool for representing reaction spaces.

**Table 4:** Comparison of qualitative evaluation of reaction embedding spaces related to ReactionT5, Morgan Fingerprint, and T5Chem

Method	Product	Retrosynthesis	Yield
Morgan Fingerprint	2.90 $\pm$ 8.10	32.08 $\pm$ 26.24	0.807 $\pm$ 0.035
T5Chem	25.55 $\pm$ 15.99	34.57 $\pm$ 28.10	**0.845 **$\pm$** 0.046**
ReactionT5	**2.76** $\pm$ **7.60**	**31.60** $\pm$ **28.22**	0.840 $\pm$ 0.020

This table presents the average Levenshtein distance for product and retrosynthesis prediction, as well as R^2^ for yield prediction

## Conclusions

In this study, we developed ReactionT5, a chemical reaction foundation model. The main contribution of this research lies in the use of reaction pre-training on a large-scale reaction database, which is the ORD. Through two-stage pre-training—compound and reaction pre-training—ReactionT5 achieved superior performance in benchmarks for product prediction, retrosynthesis, and yield prediction. Notably, ReactionT5 outperformed existing models that were fine-tuned on complete target datasets, even when fine-tuned on smaller datasets. These results suggest that pre-training on an extensive reaction database yields promising models capable of performing well in scenarios with limited training data or domain shifts. Visualization of the ReactionT5 embeddings revealed that reactions with similar features, such as mechanisms, reactant structures, and reagent types, clustered together.

Future work will focus on improving the scalability of the models and the underlying data. Although we chose a model size based on the currently available ORD dataset, the law of neural scaling suggests that expanding our training dataset through continued data collection could yield a better-performing model [34]. Additionally, multi-task learning approaches can be integrated into ReactionT5’s training to leverage complementary information from multiple tasks and enhance the model's overall performance [10]. Although this study focused only on publicly available data, we believe that our publicly available ReactionT5 can be effectively applied to real chemical applications by fine-tuning the model using private in-house experimental data. Finally, by integrating active learning into our method, chemists can efficiently probe underexplored regions of the reaction space, which leads to the development of more accurate reaction prediction models that reduce experimental trial-and-error and ultimately accelerate the discovery of novel organic transformations.

## Supplementary Information

Additional file 1.

## Data Availability

All our code is openly available at GitHub (https://github.com/sagawatatsuya/ReactionT5v2), enabling users to easily reproduce the results of this research, fine-tune the models on their in-house datasets, or leverage the pre-trained models from Hugging Face (https://huggingface.co/collections/sagawa/reactiont5-67dbe0550cbb6886a85e348b). We also provide all the datasets used in this study, as well as the parameters for each trained model.
