# Supplementary material for: ReactionT5: a pre-trained transformer model for accurate chemical reaction prediction with limited data
Source: J Cheminform. 2025 Aug 19;17:126. doi: 10.1186/s13321-025-01075-4 (PMC12366004; doi:10.1186/s13321-025-01075-4)
Supplement: Supplementary file 1 — Additional file 1. [file 13321_2025_1075_MOESM1_ESM.docx]

Supplementary data


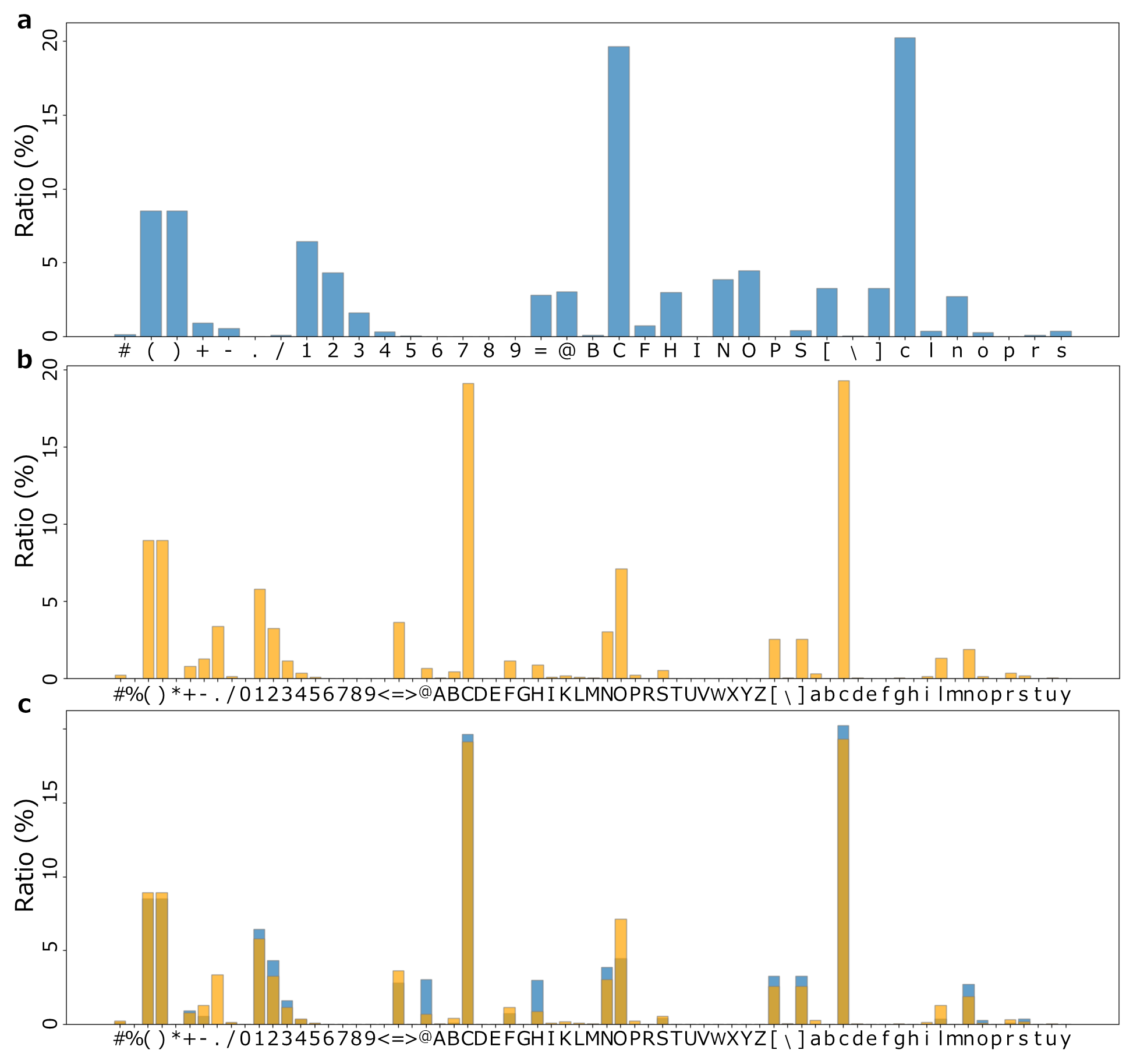
Fig. S1: Character frequency distributions in the compound pre-training dataset and the reaction database. (a) Character frequencies in the compound dataset, (b) Character frequencies in the reaction database, (c) Overlaid comparison of the two distributions to visualize differences. The reaction database exhibits a broader and more diverse character set compared to the compound dataset, including metal and inorganic elements essential for catalytic and organometallic reactions. A chi-squared test conducted on the character counts revealed a statistically significant difference between the two distributions (*p* < 2.2e-16), confirming the vocabulary mismatch and motivating the introduction of additional tokens in our tokenizer design.

Table S1: Dataset statistics.

|  | #sample | Average molecular weight | Average token length |
| --- | --- | --- | --- |
| ZINC20 [1] | 22,992,522 | 337.05 | 39.55 |
| USPTO_MIT [2] | 479,035 | 632.30 | 66.00 |
| USPTO_50k [3] | 50,037 | 371.55 | 40.16 |
| C-N cross-coupling reaction dataset [4] | 3,955 | 1,884.23 | 199.12 |
| ORD [5] (product prediction) | 1,491,293 | 708.53 | 76.11 |
| ORD (retrosynthesis prediction) | 1,491,293 | 355.75 | 38.14 |
| ORD (yield prediction) | 422,192 | 1,036.24 | 122.00 |

The “average molecular weight” and “average token length” represent the mean molecular weight and token length of the input sequences, respectively, as calculated across the dataset.


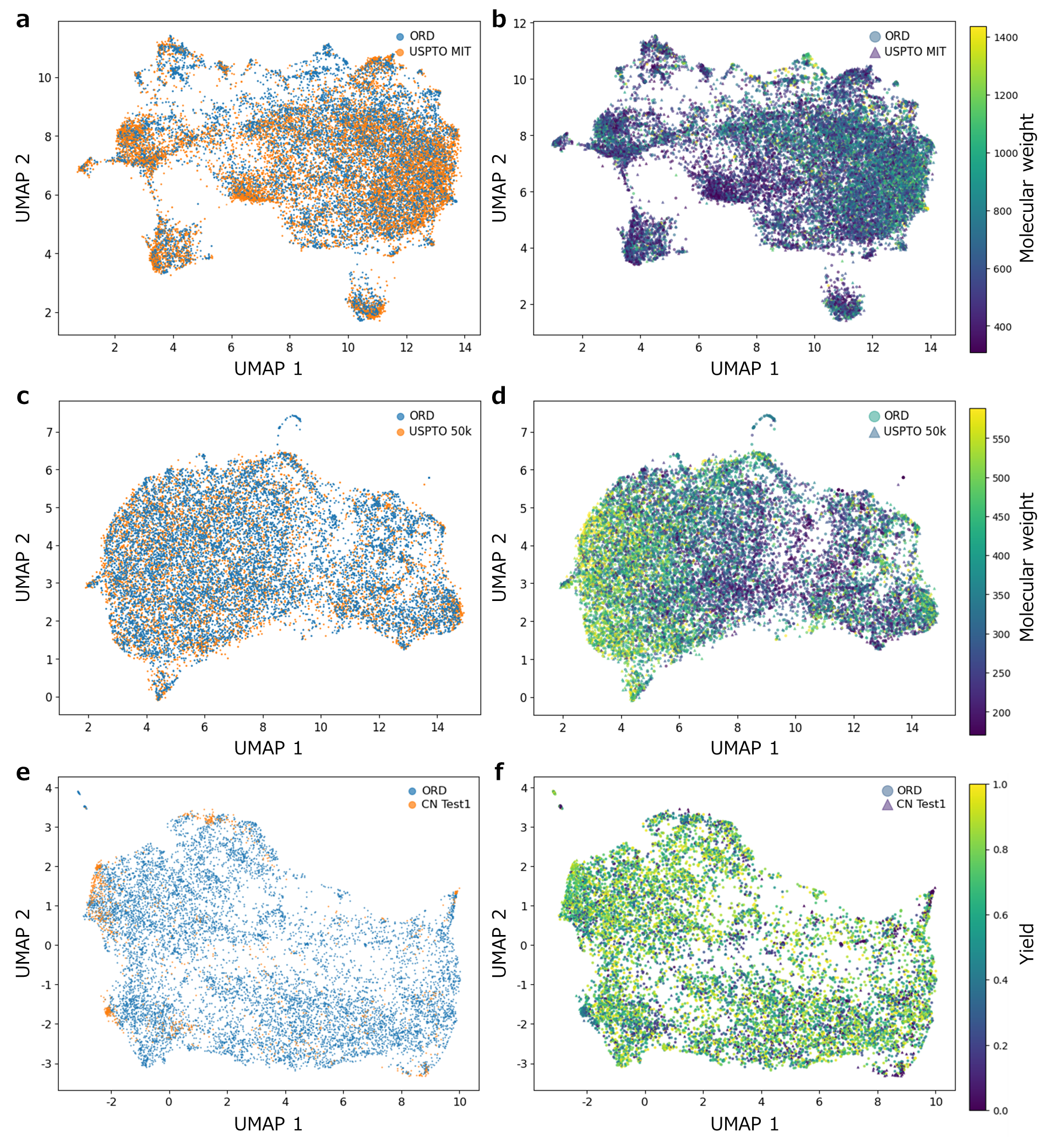
Fig. S2: Visualization of T5chem’s [6] reaction embeddings for product prediction (a and b), retrosynthesis prediction (c and d), and yield prediction (e and f). Input sequences for each reaction were encoded into hidden vectors and dimensionally reduced using UMAP. The left panels (a, c, and e) display the reaction space colored by dataset type, whereas the right panels (b, d, and f) depict variations based on either the molecular weight of input sequences or the reaction yield.


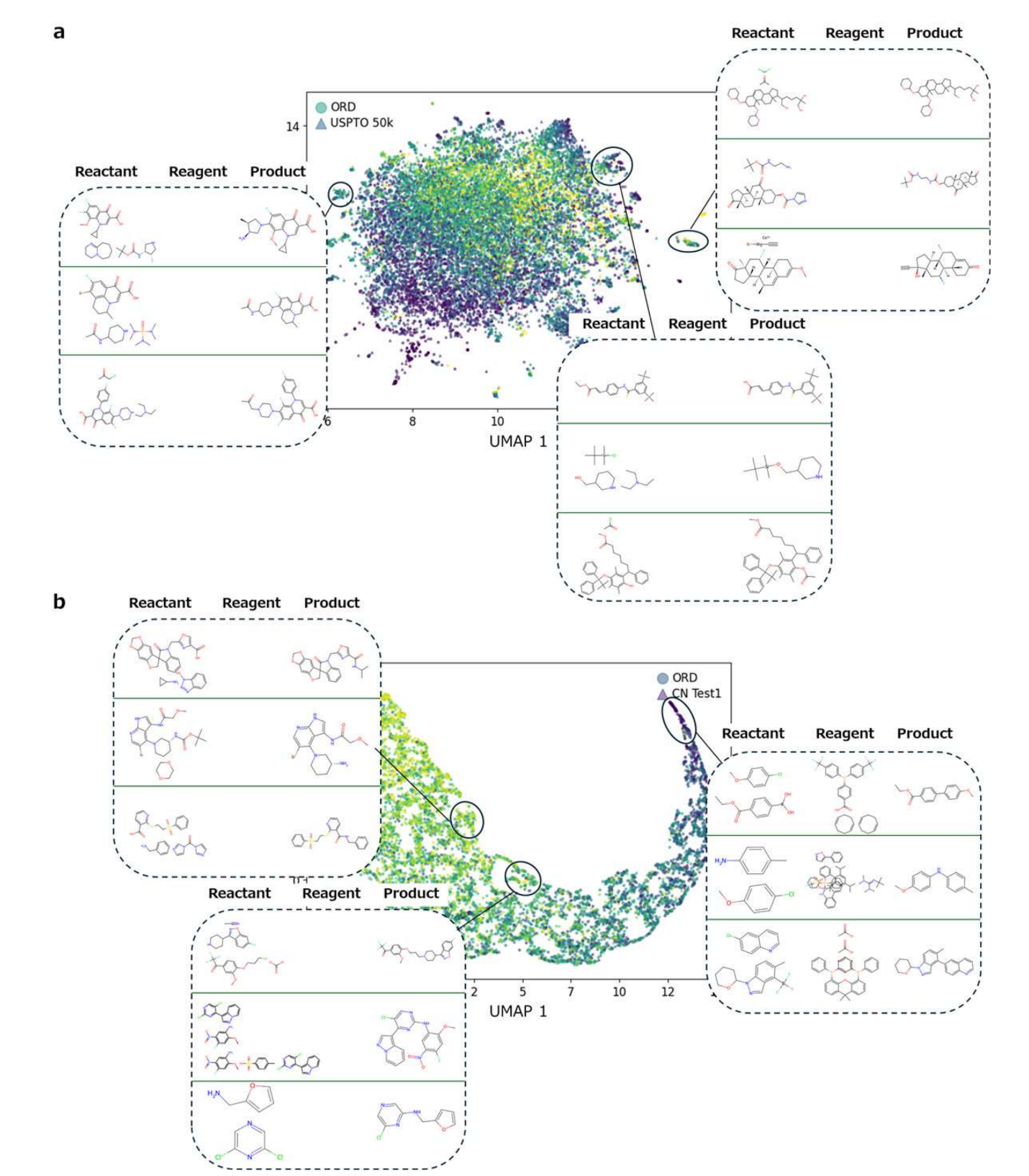


Fig. S3: Qualitative analysis of ReactionT5’s understanding of the reaction space in retrosynthesis prediction (a) and yield prediction (b) tasks. (a) Reactions on the left represent nucleophilic substitution reactions between halogenated hydrocarbons and nitrogen. Reactions on the right involve compounds with a steroid backbone, whereas those at the top include silicon-containing compounds. (b) Reactions on the left involve an amide bond. The reactions in the center are nucleophilic substitution reactions between a halogenated hydrocarbon and nitrogen. The reactions on the right are ones in which a phosphorus-containing compound is used as a reagent.


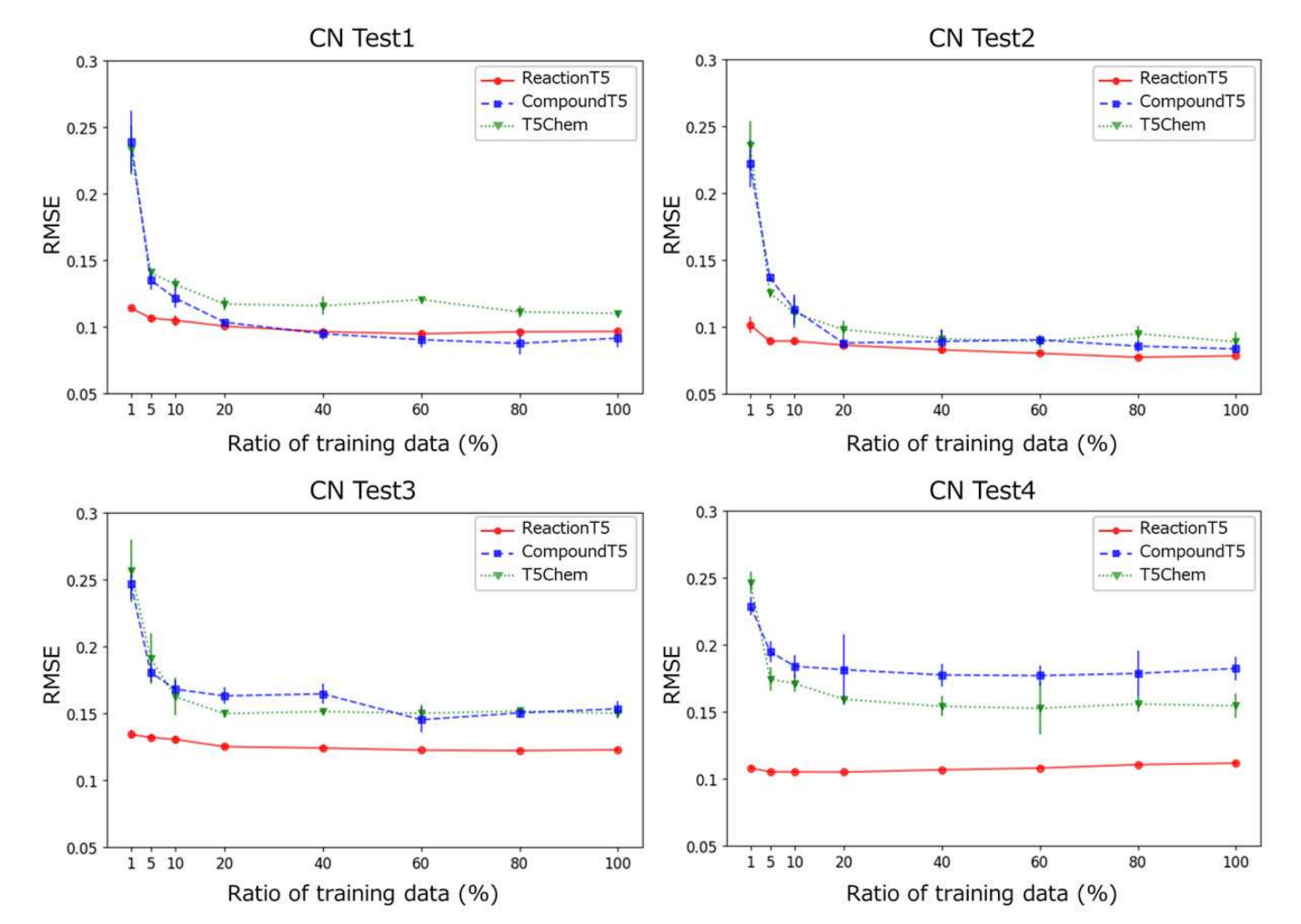
Fig. S4: RMSE for each model in yield prediction when fine-tuned with limited reaction data. The RMSE values are shown for models fine-tuned with various fractions of each dataset’s training sets. This figure shows the results of evaluating the experiment in Fig. 3 using different metrics.


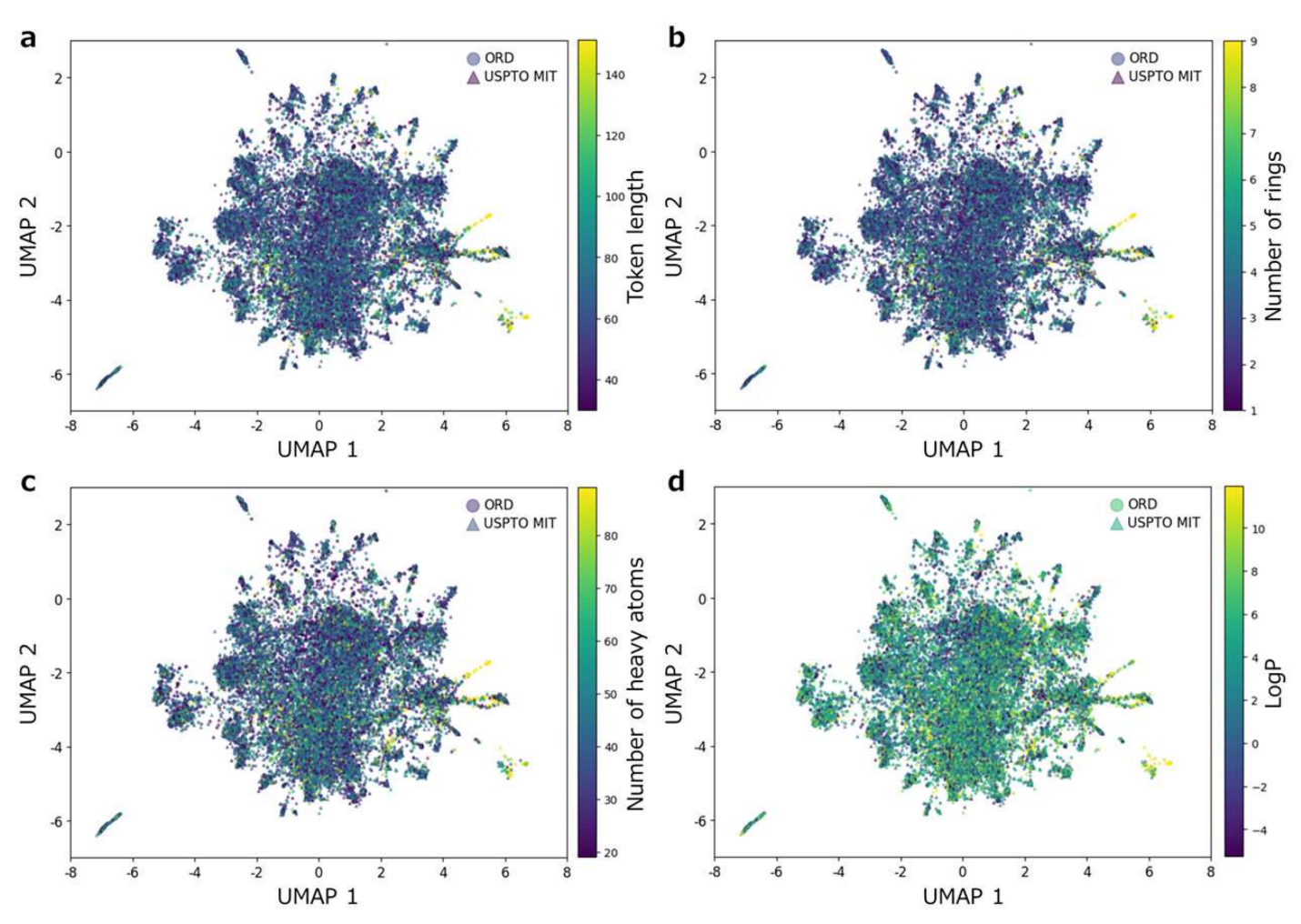
Fig. S5: Visualization of ReactionT5’s reaction spaces for product prediction Fig. 4 (a), which is colored with different molecular properties. Each reaction is colored based on its input’s (a) token length, (b) number of rings, (c) number of heavy atoms, and (d) LogP.


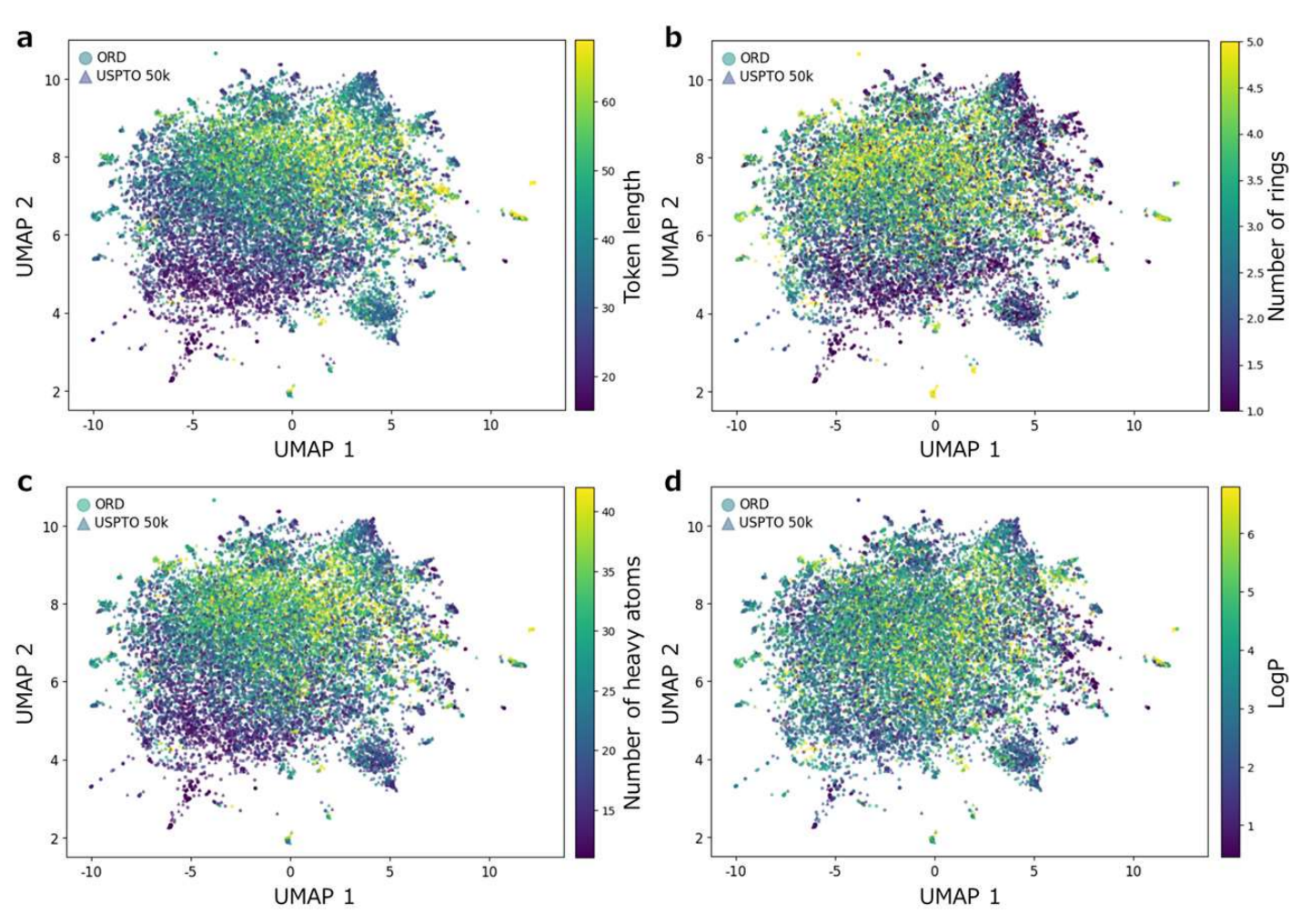


Fig. S6: Visualization of ReactionT5’s reaction space for retrosynthesis prediction Fig 4 (c), which colored with different molecular properties. Each reaction is colored based on its input’s (a) token length, (b) number of rings, (c) number of heavy atoms, and (d) LogP.


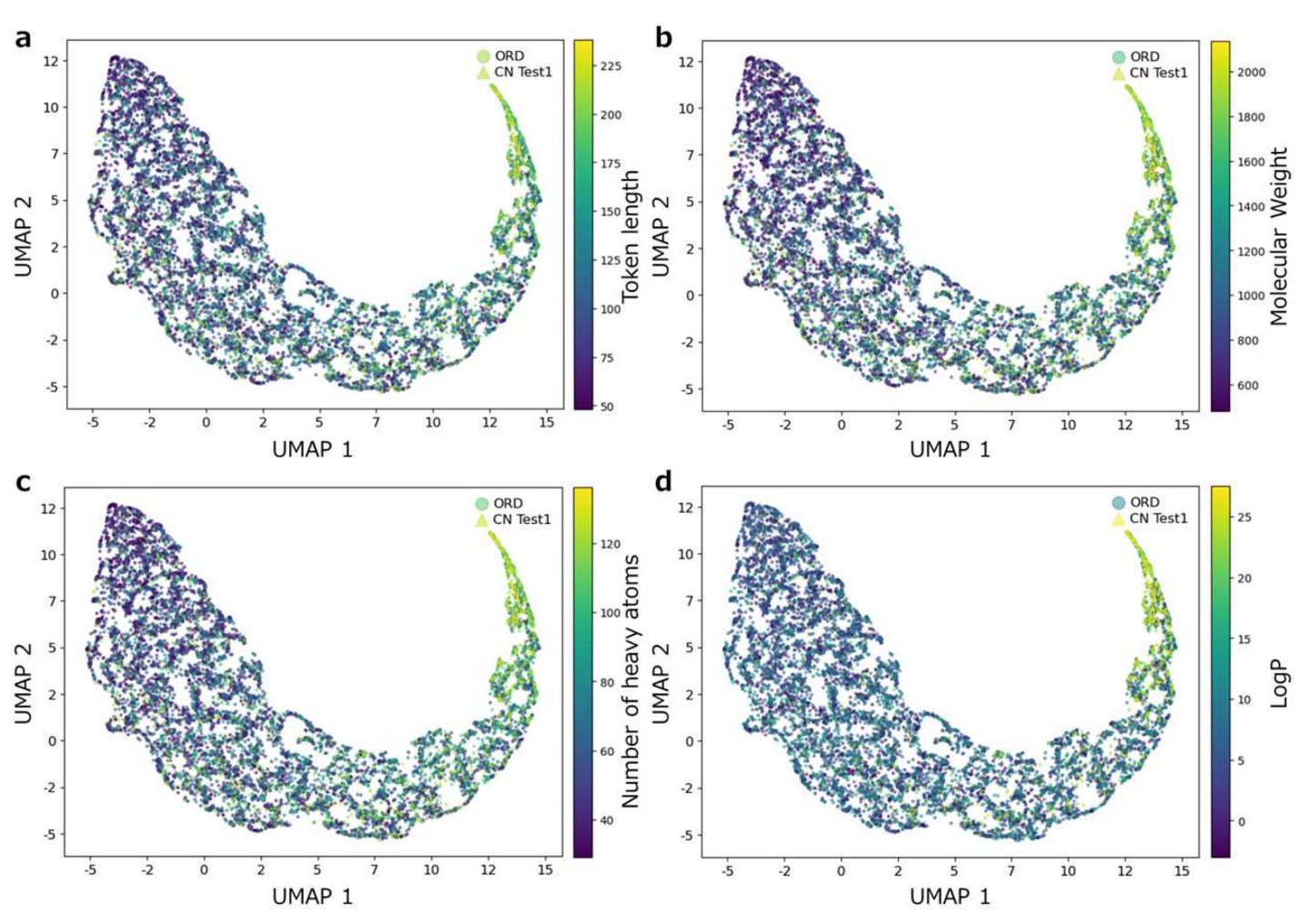
Fig. S7: Visualization of ReactionT5’s reaction space for retrosynthesis prediction in the same way as in Fig. 4 (f), except that it is colored according to different molecular properties than in Fig. 4 (f). Each reaction is colored based on its input’s (a) token length, (b) molecular weight, (c) number of heavy atoms, and (d) LogP.


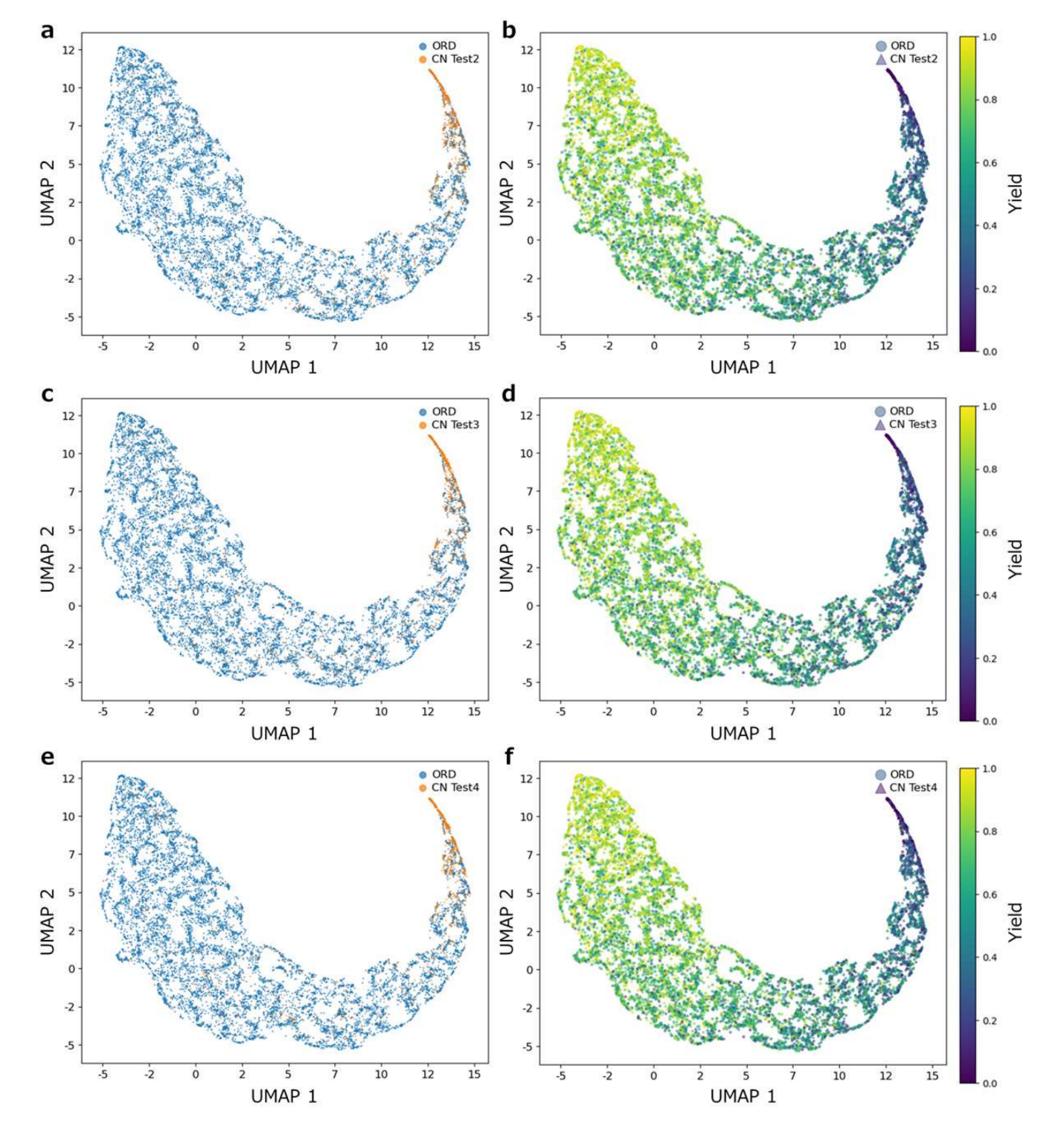
Fig. S8: Visualization of the reaction space captured by ReactionT5 for yield prediction. The left panels (a, c, and e) display the reaction space colored by dataset type, while the right panels (b, d, and f) depict variations based on reaction yield. Fig. 4 (e, f) visualizes Test 1 of the C-N cross-coupling reactions dataset, and here we show figures for Test 2 (a, b), Test 3 (c, d), and Test 4 (e, f).


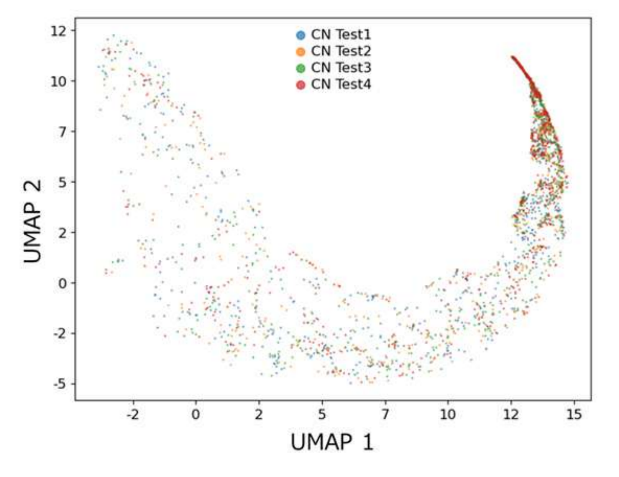


Fig. S9: Visualization of the reaction space captured by ReactionT5 for yield prediction. Reactions from Test 1-4 in palladium-catalyzed Buchwald-Hartwig C-N cross-coupling reactions dataset are visualized in the same figure, i.e., an overlay of the points corresponding to the reactions of Tests 1-4 in Fig. 4(e) and Fig. S8(a, c, e).

Table S2: Comparison of model performance on the Suzuki-Miyaura coupling dataset

|  | R^2^ | RMSE |
| --- | --- | --- |
| Yield-BERT [7] | 0.81 ± 0.01 | - |
| CompoundT5 | 0.844 ± 0.014 | 0.113 ± 0.005 |
| ReactionT5 | 0.835 ± 0.009 | 0.116 ± 0.003 |
| ReactionT5 (zero-shot) | 0.539 ± 0.018 | 0.195 ± 0.003 |

Performance comparison of yield prediction models evaluated on the Suzuki-Miyaura coupling dataset provided by Perera *et al* [8]. We used 10 different random splits of the dataset into training and validation datasets (70/30 split). ReactionT5 (zero-shot) is evaluated without fine-tuning on this Suzuki-Miyaura coupling dataset. R^2^ and RMSE were used as evaluation metrics. Both CompoundT5 and ReactionT5 outperformed Yield-BERT, with CompoundT5 achieving slightly better performance than ReactionT5.

Table S3: Effect of token extension on product prediction performance

|  | Top-1 | Top-2 | Top-3 | Top-5 | Invalidity |
| --- | --- | --- | --- | --- | --- |
| With token extension | 81.09 ± 0.08 | 86.19 ± 0.03 | 87.70 ± 0.03 | 89.05 ± 0.01 | 3.30 ± 0.05 |
| Without token extension | 76.35 ± 0.06 | 80.67 ± 0.03 | 81.91 ± 0.02 | 83.04 ± 0.02 | 11.25 ± 0.41 |

Performance comparison of the ReactionT5 model with and without token extension on the product prediction task using the ORD test set. The metrics include Top-k accuracy (k = 1, 2, 3, 5) and the Top-5 SMILES invalidity rate. Token extension significantly improved prediction accuracy (Top-1: *p* = 6.17 × 10^-7^) and reduced invalid outputs (*p* = 1.10 × 10^-3^), demonstrating the utility of vocabulary adaptation for reaction-specific data.

Table S4: Effect of token extension on yield prediction performance

|  | R^2^ | RMSE |
| --- | --- | --- |
| With token extension | 0.293 ± 0.003 | 0.221 ± 0.001 |
| Without token extension | 0.287 ± 0.005 | 0.222 ± 0.001 |

Performance comparison of the ReactionT5 model with and without token extension on the yield prediction task using the ORD test set. While slight improvements were observed in both R^2^ and RMSE when token extension was applied, Welch’s t-test for the difference in R^2^ yielded a *p*-value of 0.257, indicating no statistically significant difference.

Table S5: Categories of Added Tokens for Tokenizer Extension

| Category | Tokens |
| --- | --- |
| Metal | Ag, Al, Au, Ba, Ca, Cu, Fe, Hg, K, Li, Mg, Mn, Mo, Na, Nd, Ni, Pb, Pd, Pt, Re, Rh, Ru, Sm, Ta, Ti, Tl, W, Yb, Zn |
| Nonmetal | Ar, As, Bi, Cl, Ge, P, Sb, Si |
| Number | 6, 7, 8 |
| Special character | ., <, >, e, p |

List of the 47 additional tokens introduced to extend the tokenizer vocabulary for the reaction database. The tokens are grouped by category, including metal elements, nonmetals, numbers, and special characters such as punctuation and auxiliary symbols.

Table S6: Effect of SMILES augmentation on product prediction performance

|  | Top-1 | Top-2 | Top-3 | Top-5 | Invalidity |
| --- | --- | --- | --- | --- | --- |
| canonical | 81.09 ± 0.08 | 86.19 ± 0.03 | 87.70 ± 0.03 | 89.05 ± 0.01 | 3.30 ± 0.05 |
| permuted | 81.14 ± 0.01 | 86.28 ± 0.02 | 87.74 ± 0.00 | 89.07 ± 0.02 | 3.39 ± 0.03 |
| randomized | 80.69 ± 0.14 | 85.97 ± 0.10 | 87.49 ± 0.07 | 88.89 ± 0.03 | 3.03 ± 0.10 |
| permuted, randomized | 80.78 ± 0.10 | 86.01 ± 0.07 | 87.57 ± 0.03 | 88.97 ± 0.03 | 3.06 ± 0.07 |

Performance comparison of product prediction models pre-trained on the ORD dataset with and without SMILES-based data augmentation. For each augmentation setting, 20% of the training dataset were modified using one of the following strategies: permuted (randomly shuffling the order of reactants), randomized (using randomly uncanonicalized SMILES), or both. All models were pre-trained for 20 epochs and evaluated on the ORD test using three different random seeds. While slight variations in accuracy and invalidity were observed, the results indicate that SMILES augmentation did not consistently improve prediction performance.

**References**

1. Irwin JJ et al (2020) ZINC20—A free ultralarge-scale chemical database for ligand discovery. J Chem Inf Model 60:6065–6073. <https://doi.org/10.1021/acs.jcim.0c00675>

2. [Jin W, Coley CW, Barzilay R, Jaakkola T (2017) Predicting organic reaction outcomes with Weisfeiler-Lehman network. Adv Neural Inf Process Syst:2607–2616](http://paperpile.com/b/Ja51lW/BT3a)

3. [Liu B et al (2017) Retrosynthetic reaction prediction using neural sequence-to-sequence models. ACS Cent Sci 3:1103–1113. <https://doi.org/10.1021/acscentsci.7b00303>](http://paperpile.com/b/Ja51lW/Hk0P)

4. [Ahneman DT, Estrada JG, Lin S, Dreher SD, Doyle AG (2018) Predicting reaction performance in C-N cross-coupling using machine learning. Science 360:186–190. <https://doi.org/10.1126/science.aar5169>](http://paperpile.com/b/Ja51lW/03B4)

5. [Kearnes SM et al (2021) The open reaction database. J Am Chem Soc 143:18820–18826. <https://doi.org/10.1021/jacs.1c09820>](http://paperpile.com/b/Ja51lW/QyZi)

6. [Lu J, Zhang Y (2022) Unified deep learning model for multitask reaction predictions with explanation. J Chem Inf Model 62:1376–1387. <https://doi.org/10.1021/acs.jcim.1c01467>](http://paperpile.com/b/Ja51lW/92QM)

7. [Schwaller P, Vaucher AC, Laino T, Reymond J-L (2021) Prediction of chemical reaction yields using deep learning. Mach Learn.: Sci Technol 2:015016. <https://doi.org/10.1088/2632-2153/abc81d>](http://paperpile.com/b/Ja51lW/BitR)

8. Perera D et al (2018) A platform for automated nanomole-scale reaction screening and micromole-scale synthesis in flow. Science 259:492–434. https://doi.org/10.1126/science.aap9112
